# En Route to a Chiral Melanin: The Dynamic “From-Imprinted-to-Template” Supramolecular Role of Porphyrin Hetero-Aggregates During the Oxidative Polymerization of L-DOPA

**DOI:** 10.3389/fchem.2020.616961

**Published:** 2020-12-21

**Authors:** Massimiliano Gaeta, Rosalba Randazzo, Valentina Villari, Norberto Micali, Alessandro Pezzella, Roberto Purrello, Marco d'Ischia, Alessandro D'Urso

**Affiliations:** ^1^Dipartimento di Scienze Chimiche, Università degli Studi di Catania, Catania, Italy; ^2^Consiglio Nazionale delle Ricerche-IPCF Istituto per i Processi Chimico-Fisici, Messina, Italy; ^3^Department of Physics “Ettore Pancini,” University of Naples “Federico II”, Naples, Italy; ^4^Department of Chemical Sciences, University of Naples “Federico II”, Naples, Italy

**Keywords:** eumelanin, DOPA, porphyrin, supramolecular aggregates, circular dichroism

## Abstract

Chiral porphyrin hetero-aggregates, produced from meso-tetrakis(4-N-methylpyridyl) porphyrin H_2_T4 and copper(II) meso-tetrakis(4-sulfonatophenyl)porphyrin CuTPPS by an imprinting effect in the presence of L-3,4-dihydroxyphenylalanine (L-DOPA), are shown herein to serve as templates for the generation of chiral structures during the oxidative conversion of the amino acid to melanin. This remarkable phenomenon is suggested to involve the initial role of L-DOPA and related chiral intermediates like dopachrome as templates for the production of chiral porphyrin aggregates. When the entire chiral pool from DOPA is lost, chiral porphyrin hetero-aggregate would elicit axially chiral oligomer formation from 5,6-dihydroxyindole intermediates in the later stages of melanin synthesis. These results, if corroborated by further studies, may open unprecedented perspectives for efficient strategies of asymmetric melanin synthesis with potential biological and technological applications.

## Introduction

**Figure 1:**
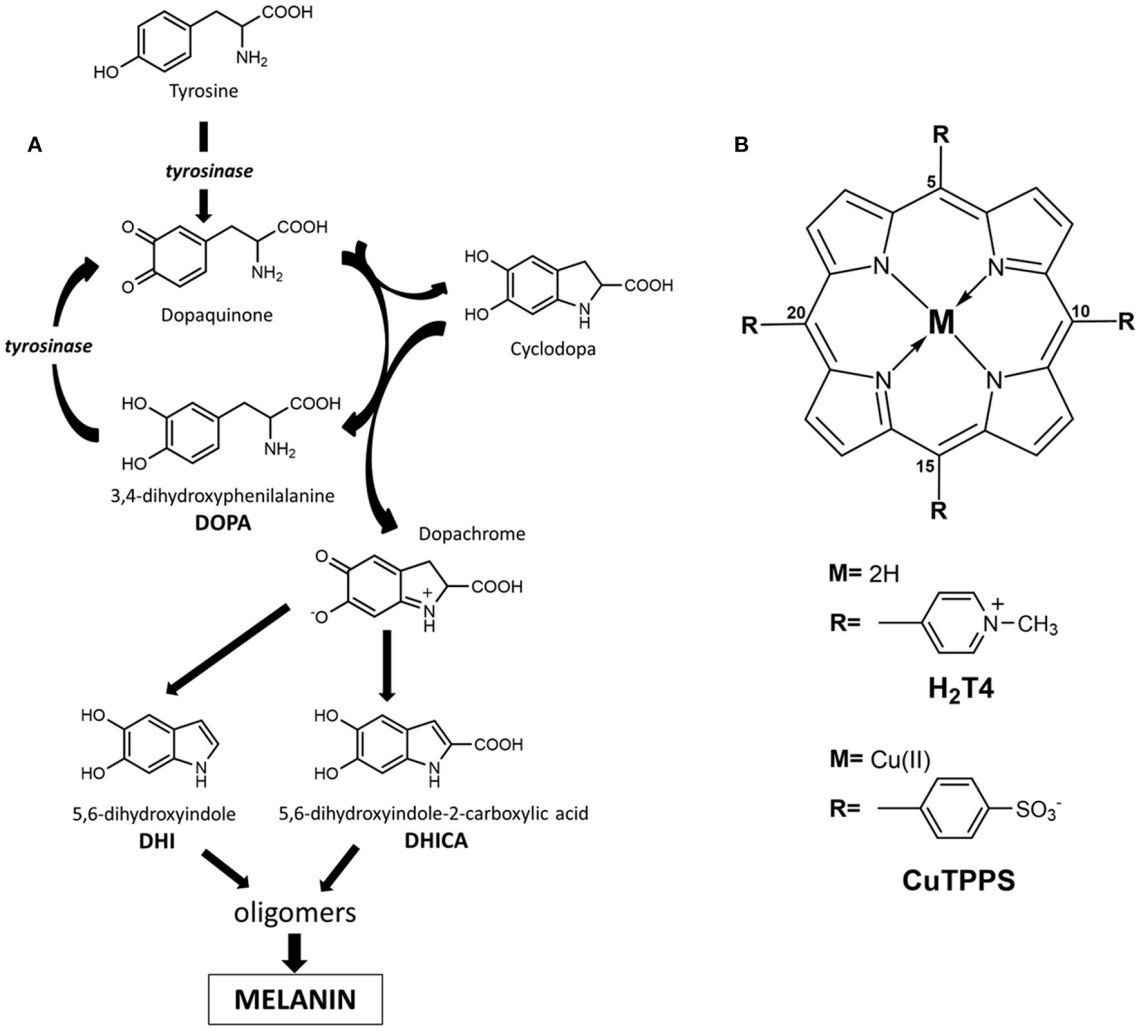
**(A)** Schematic illustration of dihydroxyphenylalanine (DOPA) oxidative polymerization to melanin (highlighted is the generation of L-3,4-dihydroxyphenylalanine (L-DOPA) by redox cycling between leucodopachrome and dopaquinone). **(B)** Molecular structures of porphyrinoids used, 5,10,15,20-tetrakis(4-N-methylpyridyl)porphyrin tetrachloride salt (H_2_T4) and copper(II) 5,10,15,20-tetrakis(4-sulfonatophenyl)porphyrin tetrasodium salt (CuTPPS).

L-3,4-Dihydroxyphenylalanine (L-DOPA; Figure 1A) is an aromatic amino acid produced in various organisms by the oxidative modification of L-tyrosine (Raper, 1927; Mason and Wright, 1949; Haneda and Watanabe, 1971; Prota, 1995; Ito, 2003; Slominski et al., 2012; Marchiosi et al., 2020). In the skin and other organs, like the ink sac of cephalopods, L-DOPA is an early intermediate in the synthesis of black eumelanin pigments from L-tyrosine by the action of the copper enzyme tyrosinase (Slominski et al., 2004; Ito and Wakamatsu, 2007; Simon et al., 2009; Della Vecchia et al., 2013; d'Ischia et al., 2014). *In vivo*, the process involves the oxidation of tyrosine to dopaquinone followed by intramolecular cyclization to an indoline intermediate, termed leucodopachrome or cyclodopa, which can enter a redox cycle by exchanging electrons with dopaquinone to produce L-DOPA and L-dopachrome. The latter, then, undergoes isomerization with or without decarboxylation and loss of the chiral center to give 5,6-dihydroxyindole (DHI) and/or 5,6-dihydroxyindole-2-carboxylic acid (DHICA), respectively (Pezzella et al., 1996; Edge et al., 2006; Ito and Wakamatsu, 2007, 2008; Ito et al., 2011; d'Ischia et al., 2013; Panzella et al., 2018). The oxidative polymerization of these latter intermediates leads to the deposition of black insoluble melanin polymers (Figure 1A). So far, knowledge of the intrinsic chiroptical features of melanin polymers is scant, and little attention has been paid to the possible generation of chiral structures under *in vivo* or *in vitro* conditions. The only evidence for the possible occurrence of chirality in melanins is due to the demonstration that oligomers from DHICA may display atropisomerism caused by hindered rotation about interunit bonds (Pezzella et al., 2003). However, current information on the chirality of DHICA oligomers during the polymerization process remains limited.

Recently, a systematic investigation of the effect of porphyrin aggregation on melanin synthesis was undertaken, exploiting the well-known tendency of porphyrins to interact with amino acids (Angelini et al., 2005; Uemori et al., 2012; Charalambidis et al., 2016; Gaeta et al., 2016; Rananaware et al., 2016; Ryu et al., 2018) and polymeric chains (Borovkov et al., 2002a,b; De Luca et al., 2010; Occhiuto et al., 2015; Zhao et al., 2015; Gaeta et al., 2018), with a view to generating new bioinspired functional materials with tailored optical and chiral properties. Water-soluble porphyrins maintain their tendency to aggregate owing to the hydrophobic aromatic macrocycle, whereby binding suitable functional groups to the porphyrin ring may allow to realize self-assembled porphyrin systems in aqueous solution. Although supramolecular arrangements of achiral porphyrins in aqueous solution result in achiral supramolecular structures, chiral aggregates of porphyrins can be formed in the presence of chiral templates (Bellacchio et al., 1998; Onouchi et al., 2006; Toyofuku et al., 2007; Lauceri et al., 2008; Helmich et al., 2010).

Noteworthy, as a consequence of extensive network of interactions (electrostatic, solvophobic, etc.) that trap porphyrin aggregates in a quite deep local energy minimum ensuring kinetic inertia, the porphyrin supramolecular assembly is able to *memorize* the chiral information imprinted by the chiral template in aqueous solution (Mammana et al., 2007; Gaeta et al., 2016). In this context, porphyrin hetero-aggregates (built by opposite-charged porphyrins) proved to be an ideal system to store chiral information and may offer the possibility of designing switch of memory (Mammana et al., 2007). In this work, we show that porphyrin hetero-aggregates made up of 5,10,15,20-tetrakis(4-N-methylpyridyl)porphyrin H_2_T4 (Figure 1B) and copper(II) 5,10,15,20-tetrakis(4-sulfonatophenyl)porphyrin CuTPPS (Figure 1B) can drive the oxidative polymerization of DOPA to melanins with the unexpected generation of asymmetric structures.

## Materials and Methods

5,10,15,20-Tetrakis(4-N-methylpyridyl)porphyrin tetrachloride salt (H_2_T4) and copper(II) 5,10,15,20-tetrakis(4-sulfonatophenyl)porphyrin tetrasodium salt (CuTPPS) were purchased from Mid-Century Company and used without further purification. Porphyrin mother solutions (about 4 × 10^−4^ M, stored in the dark at room temperature) were prepared dissolving the solid in ultrapure water obtained from Elga Purelab Flex system by Veolia. Then, the concentration was checked by spectrophotometric methods in water solution at neutral pH by means of their molar extinction coefficients at maximum of the Soret band: λ_max_(H_2_O) = 423 nm (ε = 224,000 M^−1^cm^−1^) for H_2_T4 and λ_max_(H_2_O) = 412 nm (ε = 416,000 M^−1^cm^−1^) for CuTPPS.

The phosphate buffered saline (PBS) tablets were purchased from Sigma-Aldrich Company, and the stock solution was prepared by dissolving one tablet in 200 ml of ultrapure water. PBS buffer (pH = 7.4) contains 10 mM of phosphate buffer sodium salt, 137 mM of sodium chloride, and 2.7 mM of potassium chloride.

L-DOPA and the respective D-enantiomer [D-3,4-dihydroxyphenylalanine (D-DOPA)] were purchased from Sigma-Aldrich Company and used without further purification. Solutions of both DOPA enantiomers were freshly prepared by solubilizing the proper amount of solid in PBS buffer in order to attain a final concentration of 0.5 mM.

The porphyrin hetero-aggregates in PBS buffer were obtained by filling with 2 ml of PBS solution a quartz cuvette (path length = 1 cm), then the proper volume of H_2_T4 mother solution was added to reach a 2 μM concentration of H_2_T4 in the sample solution. After 5 min, the amount of CuTPPS was added in order to reach again a 2 μM concentration of CuTPPS in the sample solution. After an additional 20 min, other aliquots of H_2_T4 and CuTPPS were added as illustrated before. The final work solution thus obtained was kept for 20 min before spectroscopic investigations.

The porphyrin hetero-aggregates in the presence of D- and L-DOPA were obtained by using the corresponding DOPA solution (0.5 mM in PBS) following the aforementioned procedure. In detail, the proper volume of H_2_T4 mother solution was added to D- or L-DOPA solution to reach a 2 μM concentration of H_2_T4, then after 5 min, the proper amount of CuTPPS was added to the sample solution in order to reach again a 2 μM concentration of CuTPPS. After an additional 20 min, other aliquots of H_2_T4 and CuTPPS were added as illustrated before. The final work solution thus obtained was kept for 20 min before spectroscopic investigations. For the long incubation time, each solution of DOPA and porphyrin hetero-aggregates was stored in sealed plastic cuvettes in order to limit the adhesion of both porphyrins and DOPA on the cuvette walls.

All solutions, both stock and sample solutions, are prepared in ultraclean conditions: (i) the operators wore a lab coat, hair cap, gloves, and mask during the preparation of samples and (ii) the tips of the pipettes and the plastic cells were washed three times with ultrapure water before being used.

Circular dichroism (CD) and Uv/Vis measurements were carried out at room temperature (quartz cuvette path length 1 cm) on a JASCO J-710 spectropolarimeter and JASCO V-530 spectrophotometer, respectively. Linear dichroism (LD) measurements were carried out on a modified JASCO J-500A spectropolarimeter (Micali et al., 2015) after proper calibration with an oriented polarizer. Linear birefringence of the instrument optics was also measured in order to evaluate the cross-talk contribution to CD.

## Results and Discussions

In PBS buffer (pH = 7.4), the formation of porphyrin hetero-aggregates from equimolar amounts of tetra-cationic H_2_T4 and tetra-anionic CuTPPS was apparent by both the hypochromic effect and band broadening in the Soret region, as reported in UV/Vis spectrum (Supplementary Figure 1). In the absence of chiral inducers in solution and under ultraclean conditions, the building of achiral supramolecular structures, as expected, was denoted by zero optical activity in the porphyrin hetero-aggregate absorption region (Supplementary Figure 1 inset).

**Figure 2:**
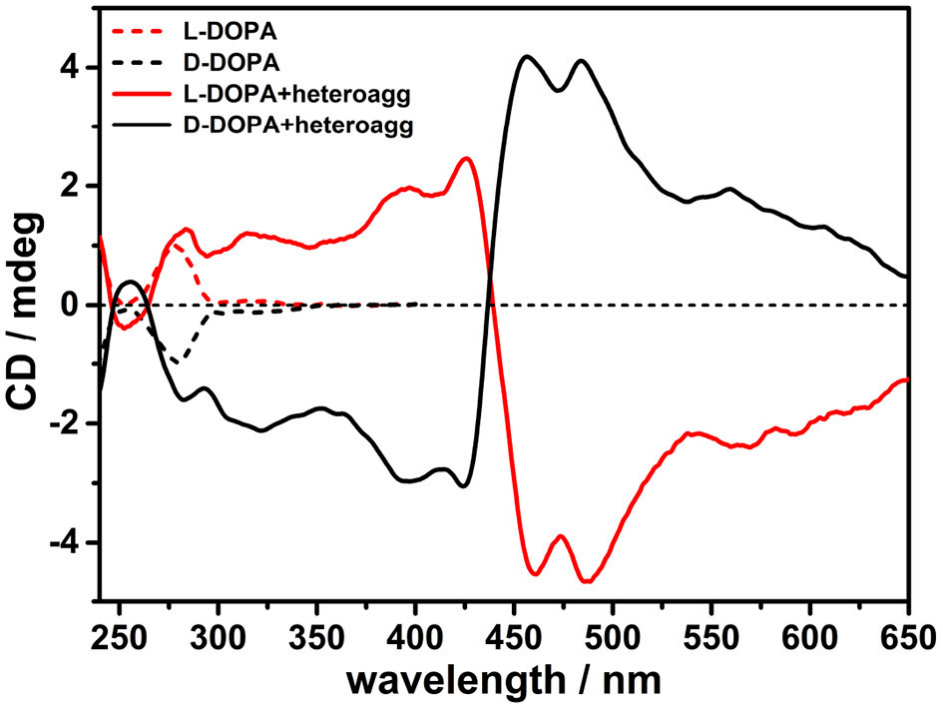
Circular dichroism (CD) spectra in phosphate buffered saline (PBS) buffer (pH = 7.4) of porphyrin hetero-aggregates [5,10,15,20-tetrakis(4-N-methylpyridyl)porphyrin tetrachloride salt (H_2_T4) = 4 μM, copper(II) 5,10,15,20-tetrakis(4-sulfonatophenyl)porphyrin tetrasodium salt (CuTPPS) = 4 μM] in the presence of L-3,4-dihydroxyphenylalanine (L-DOPA; red solid curve) and D-3,4-dihydroxyphenylalanine (D-DOPA; black solid curve) as prepared. The CD spectra for dihydroxyphenylalanine (DOPA) alone in PBS buffer are graphed in red dashed curve for L-enantiomer and in black dashed curve for D-enantiomer. In all samples, the concentration of DOPA was 0.5 mM.

The construction of chiral multicomponent systems requires precise hierarchical rules (Elemans et al., 2003), whereby to prepare chiral porphyrin hetero-aggregates, cationic porphyrin H_2_T4 (4 μM) was to be added to a PBS solution of L-DOPA (0.5 mM) followed by the anionic counterpart CuTPPS (4 μM). UV/Vis spectra confirm the formation of porphyrin hetero-aggregate, showing a hypochromic effect and broadening of the Soret bands (Supplementary Figure 2). Noteworthy in the CD spectra, both a positive single Cotton effect of L-DOPA at about 280 nm and an induced bisignate CD signal in visible region, due to chiral exciton coupling of two porphyrin chromophores in hetero-aggregates, were observed (Figure 2). Mirror image was recorded when D-DOPA was used in place of levodopa, confirming that the CD signal in the porphyrin absorption region was induced by the interaction with DOPA via a chirality transfer process (Figure 2).

**Figure 3:**
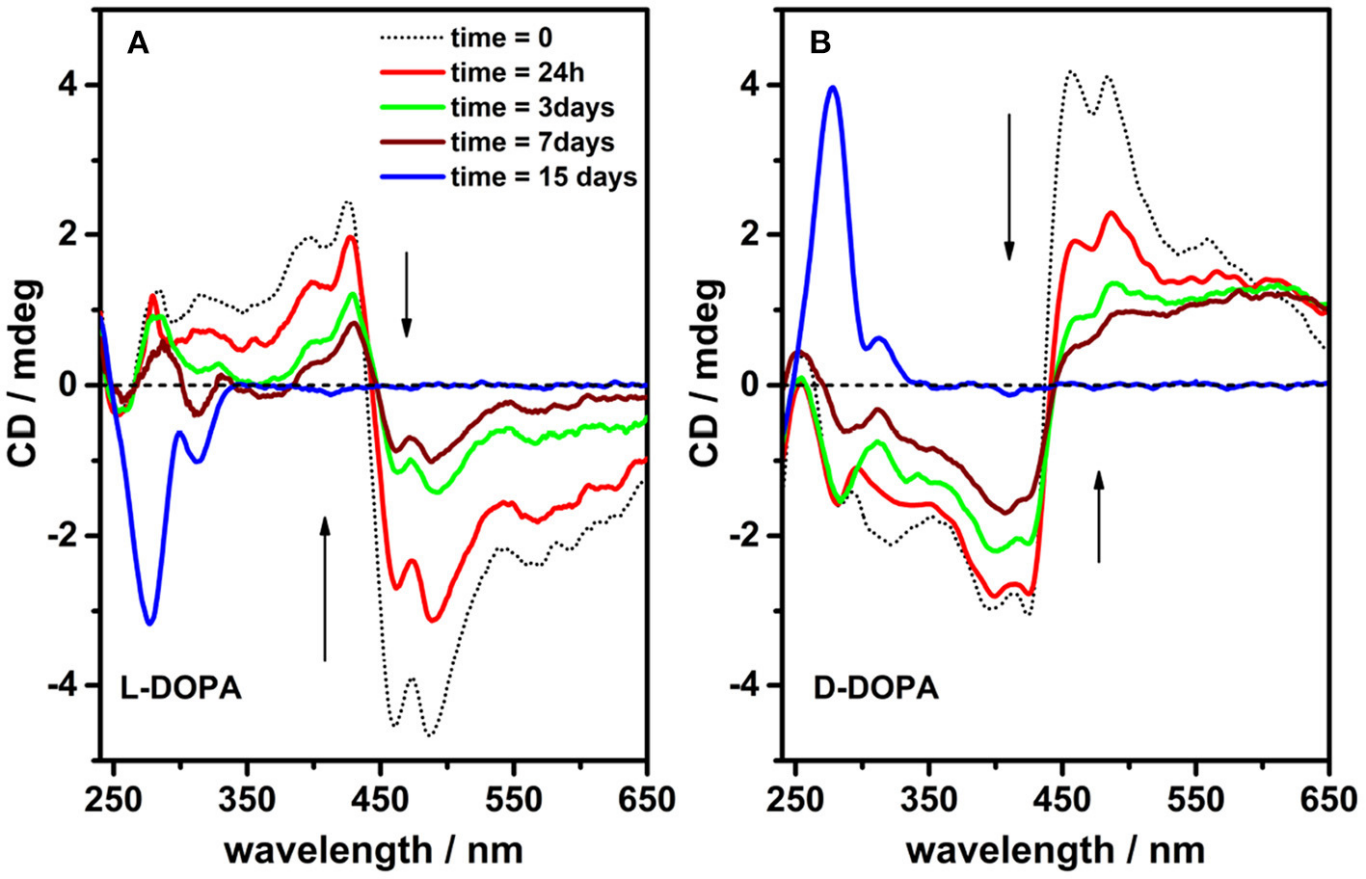
Circular dichroism spectra of incubated solutions [phosphate buffered saline (PBS) buffer, pH = 7.4] containing porphyrin hetero-aggregates [5,10,15,20-tetrakis(4-N-methylpyridyl)porphyrin tetrachloride salt (H_2_T4) = 4 μM, copper(II) 5,10,15,20-tetrakis(4-sulfonatophenyl)porphyrin tetrasodium salt (CuTPPS) = 4 μM] in the presence of L-3,4-dihydroxyphenylalanine (L-DOPA) 0.5 mM **(A)** and D-3,4-dihydroxyphenylalanine (D-DOPA) 0.5 mM **(B)** as prepared (dotted black curves) and after 24 h, 3, 7, and 15 days (red, green, wine, and blue curves in that order).

The solutions containing chiral porphyrin hetero-aggregates and single DOPA enantiomers were incubated for 2 weeks in plastic cuvettes rather than in quartz cuvettes in order to minimize the sticking of DOPA-derived melanin products onto cuvette walls. Although the oxidative polymerization of DOPA evolved slowly in PBS buffer at pH 7.4 as is usually the case with catechol and catecholamine compounds (Bernsmann et al., 2011), after 2 weeks, significant variations in the DOPA absorption and CD spectra were observed. CD and UV spectra of the sample solutions were recorded after 24 h, 3, 7, and 15 days to follow the evolution of DOPA polymerization. In comparison with the initial situation, main changes observed concerned: *i)* the loss of the induced CD signal attributed to the porphyrin hetero-aggregates and, simultaneously, *ii)* a marked change in the dichroic signal of DOPA (Figure 3, Supplementary Figure 3).

Since the chirality transfer mechanism implies a close-range contact between chiral inducer and achiral building blocks (Borovkov et al., 2001; Mammana et al., 2007; Zeng et al., 2009; Randazzo et al., 2019; Ustrnul et al., 2019), the disappearance of the induced chirality may be associated to a de-aggregation of the porphyrin hetero-aggregate owing to the polymerization of DOPA and the associated loss of chirality. In line with this conclusion, the UV/Vis spectra of incubated hetero-aggregates evolved with the growth of the CuTPPS Soret band (λ_*max*_ = 412 nm) (Supplementary Figure 3), whereas, conversely, no detectable band associated with H_2_T4 (λ_*max*_ = 423 nm) was observed (Supplementary Figure 3 inset) presumably due to embedment into the developing melanin matrix whose carboxylate residues are deprotonated (thus anionic charged) at pH value of PBS buffer. Indeed, adding acid solution to melanin precipitate (separated from the solution) in order to protonate the carboxylate residues, a band at 450 nm, ascribable to protonated form of H_2_T4, was detected (Supplementary Figure 4). These spectroscopic data suggested that the porphyrin hetero-aggregate in PBS at high ionic strength does not exhibit similar stability as previously demonstrated in water (Mammana et al., 2007; Gaeta et al., 2016). It is likely that ionic strength modulates electrostatic interactions between opposite-charged porphyrins, affecting the stability of the hetero-aggregate in PBS.

Remarkably, drastic changes in the CD signals at 450–500 nm are observed with time (1 week timescale; Figure 3), which are paired to a later increase of the signal at 280 nm (2 weeks timescale). Such profile evolution is a clear signature of the generation of asymmetric structures, likely driven by chirally imprinted porphyrin hetero-aggregates during melanin synthesis, while the original chiral information from DOPA was completely consumed because of its conversion into 5,6-dihydroxyindoles. Noteworthy, contributions from LD are negligible; however, the possibility that a component of differential scattering might affect the measurements owing to the presence of melanin particles cannot be ruled out.

**Figure 4:**
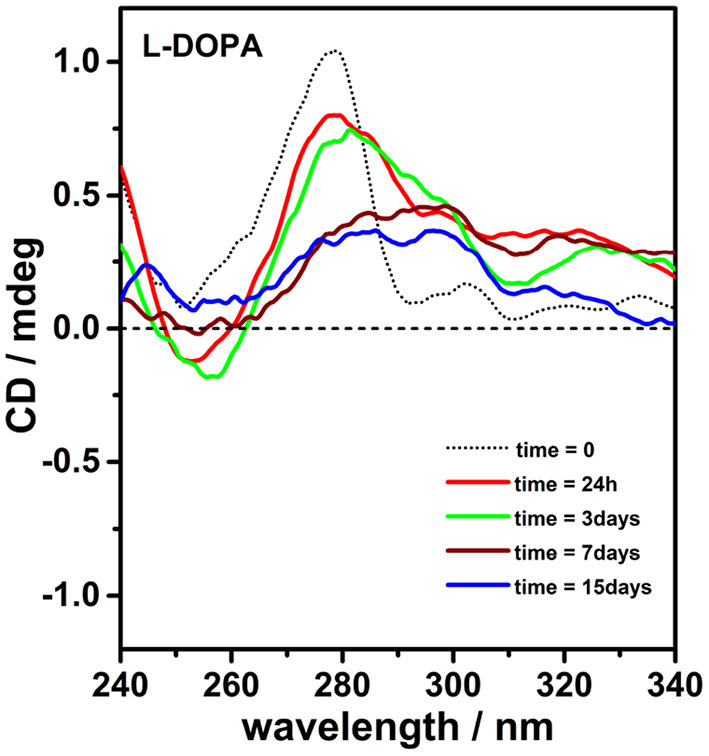
Circular dichroism spectra of L-3,4-dihydroxyphenylalanine (L-DOPA; 0.5 mM) alone in phosphate buffered saline (PBS) buffer (pH = 7.4, 298K) as prepared (dotted black curve) and after 24 h, 3, 7, and 15 days (red, green, wine, and blue curves, respectively).

To support the above conclusions, the spectroscopic behavior of L-DOPA in PBS buffer was monitored in the absence of porphyrins. The progress of the oxidative polymerization was denoted by the simultaneous decrease of the absorbance (λ_*max*_ = 280 nm) and related CD signal of L-DOPA (Figure 4, Supplementary Figure 5). After several days, the final dark solution did not display almost any residual chirality suggesting the formation of achiral melanin.

Further evidences of the role played by porphyrin hetero-aggregate as chiral templating agent of melanin oligomers have been gained performing a clear-cut experiment, reversing the order of addition of the components. In detail, we added L-DOPA to a solution of preformed achiral porphyrin hetero-aggregate (Supplementary Figure 6). After 1 week, the CD spectrum of L-DOPA looked similar to the CD spectrum of L-DOPA alone in PBS (Supplementary Figure 6 inset), confirming that chiral porphyrin hetero-aggregate plays a key role in inducing chiral melanin oligomer formation.

**Scheme 1:**
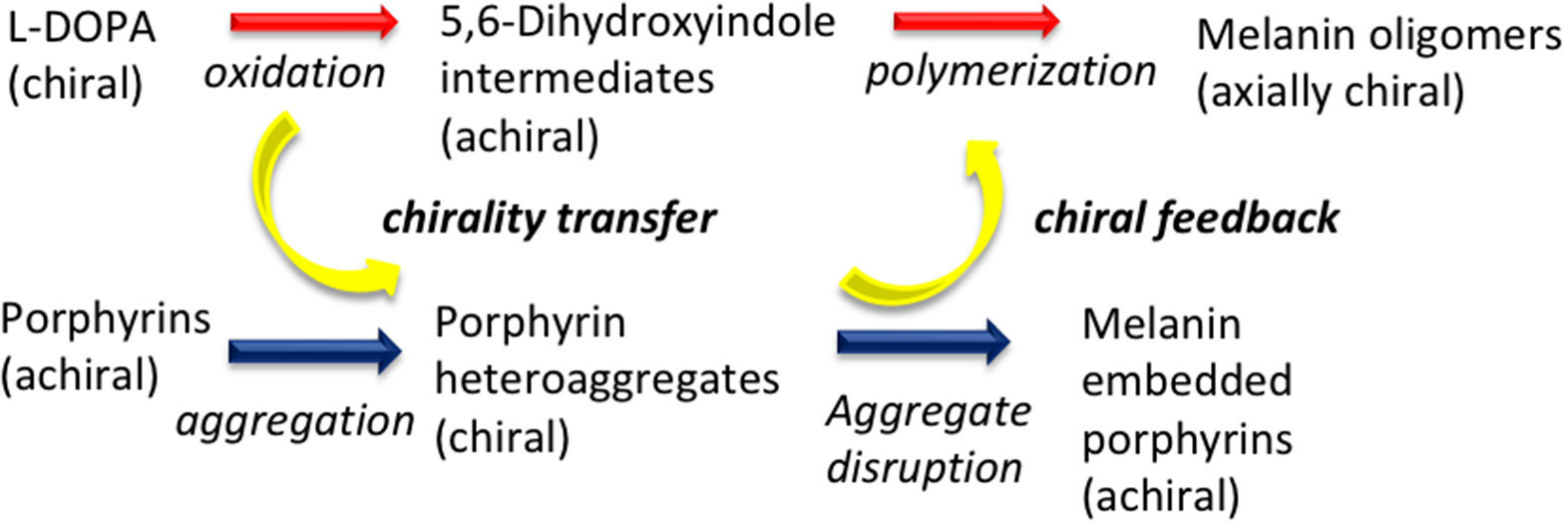
Chirality transfer dynamics in the oxidative polymerization of L-3,4-dihydroxyphenylalanine (L-DOPA) in the presence of porphyrin hetero-aggregate.

To conclude, these results disclose a rare example of temporary chiral mediation in which a chirally imprinted aggregate is decomposed while serving in turn as template for the chiral imprinting of developing oligomer aggregates from non-chiral decomposition products of a chiral precursor (Scheme 1).

These results open a new promising area of investigation on the organization of melanin pigments with applications ranging from biology and medicine to nanotechnology and material science.

## Data Availability Statement

The raw data supporting the conclusions of this article will be made available by the authors, without undue reservation.

## Author Contributions

AD'U, Md'I, AP, and RP contributed to the conception and design of the study. Experimental work was carried out by MG, RR, VV, and NM (CD, UV/Vis, LD, differential scattering) under supervision of AD'U. MG wrote the manuscript and prepared images with contributions of AD'U, Md'I, and AP. All authors participated in the analysis and discussion of obtained results.

## Conflict of Interest

The authors declare that the research was conducted in the absence of any commercial or financial relationships that could be construed as a potential conflict of interest.

## References

[B1] Angelini N. Micali N. Mineo P. Scamporrino E. Villari V. Vitalini D. (2005). Uncharged water-soluble Co(II) - Porphyrin: a receptor for aromatic α-amino acids. J. Phys. Chem. B 109, 18645–18651. 10.1021/jp052408u 16853399

[B2] Bellacchio E. Lauceri R. Gurrieri S. Scolaro L. M. Romeo A. Purrello R. (1998). Template-imprinted chiral porphyrin aggregates. J. Am. Chem. Soc. 120, 12353–12354. 10.1021/ja982089311829583

[B3] Bernsmann F. Ball V. Addiego F. Ponche A. Michel M. Gracio J. J. D. A.. (2011). Dopamine-melanin film deposition depends on the used oxidant and buffer solution. Langmuir 27, 2819–2825. 10.1021/la104981s 21332218

[B4] Borovkov V. V. Lintuluoto J. M. Inoue Y. (2001). Supramolecular chirogenesis in zinc porphyrins: mechanism, role of guest structure, and application for the absolute configuration determination. J. Am. Chem. Soc. 123, 2979–2989. 10.1021/ja0032982 11457008

[B5] Borovkov V. V. Lintuluoto J. M. Inoue Y. (2002a). Stoichiometry-controlled supramolecular chirality induction and inversion in bisporphyrin systems. Org. Lett. 4, 169–171. 10.1021/ol016870i 11796042

[B6] Borovkov V. V. Lintuluoto J. M. Sugiura M. Inoue Y. Kuroda R. (2002b). Remarkable stability and enhanced optical activity of a chiral supramolecular bis-porphyrin tweezer in both solution and solid state. J. Am. Chem. Soc. 124, 11282–11283. 10.1021/ja026884z 12236738

[B7] Charalambidis G. Georgilis E. Panda M. K. Anson C. E. Powell A. K. Doyle S.. (2016). A switchable self-assembling and disassembling chiral system based on a porphyrin-substituted phenylalanine-phenylalanine motif. Nat. Commun. 7, 1–11. 10.1038/ncomms12657 27582363 PMC5025786

[B8] De Luca G. Romeo A. Scolaro L. M. Pasternack R. F. (2010). Conformations of a model protein revealed by an aggregating CuII porphyrin: sensing the difference. Chem. Commun. 46, 389–391. 10.1039/B918433C 20066301

[B9] Della Vecchia N. F. Avolio R. Alf,è M. Errico M. E. Napolitano A. d'Ischia M. (2013). Building-block diversity in polydopamine underpins a multifunctional eumelanin-type platform tunable through a quinone control point. Adv. Funct. Mater. 23, 1331–1340. 10.1002/adfm.201202127

[B10] d'Ischia M. Napolitano A. Ball V. Chen C. T. Buehler M. J. (2014). Polydopamine and eumelanin: from structure-property relationships to a unified tailoring strategy. Acc. Chem. Res. 47, 3541–3550. 10.1021/ar500273y 25340503

[B11] d'Ischia M. Wakamatsu K. Napolitano A. Briganti S. Garcia-Borron J. C. Kovacs D.. (2013). Melanins and melanogenesis: methods, standards, protocols. Pigment Cell Melanoma Res. 26, 616–633. 10.1111/pcmr.12121 23710556

[B12] Edge R. D'Ischia M. Land E. J. Napolitano A. Navaratnam S. Panzella L.. (2006). Dopaquinone redox exchange with dihydroxyindole and dihydroxyindole carboxylic acid. Pigment Cell Res. 19, 443–450. 10.1111/j.1600-0749.2006.00327.x 16965273

[B13] Elemans J. A. A. W. Rowan A. E. Nolte R. J. M. (2003). Mastering molecular matter. Supramolecular architectures by hierarchical self-assembly. J. Mater. Chem. 13, 2661–2670. 10.1039/B304972H

[B14] Gaeta M. Oliveri I. Pietro Fragal,à M. E. Failla S. D'Urso A. Di Bella S. (2016). Chirality of self-assembled achiral porphyrins induced by chiral Zn(II) Schiff-base complexes and maintained after spontaneous dissociation of the templates: a new case of chiral memory. Chem. Commun. 52, 8518–8521. 10.1039/C6CC04018G 27291354

[B15] Gaeta M. Raciti D. Randazzo R. Gangemi C. M. A. Raudino A. D'Urso A.. (2018). Chirality enhancement of porphyrin supramolecular assembly driven by a template preorganization effect. Angew. Chemie Int. Ed. 57, 10656–10660. 10.1002/anie.201806192 29939459

[B16] Haneda K. Watanabe S. (1971). Synthesis of L-3,4-dihydroxyphenylalanine from L-tyrosine by microorganisms. Appl. Microbiol. 22, 721–722. 10.1128/AEM.22.4.721-722.1971 5130435 PMC376393

[B17] Helmich F. Lee C. C. Schenning A. P. H. J. Meijer E. W. (2010). Chiral memory via chiral amplification and selective depolymerization of porphyrin aggregates. J. Am. Chem. Soc. 132, 16753–16755. 10.1021/ja1077602 21053898

[B18] Ito S. (2003). IFPCS presidential lecture: a chemist's view of melanogenesis. Pigment Cell Res. 16, 230–236. 10.1034/j.1600-0749.2003.00037.x 12753395

[B19] Ito S. Wakamatsu K. (2007). “Chemistry of Melanins,” in The Pigmentary System: Physiology and Pathophysiology: 2nd edn, eds Nordlund J. J. Boissy R. E. Hearing V. J. King R. A. Oetting W. S. Ortonne J.-P. (Oxford: Blackwell Publishing Ltd), 282–310.

[B20] Ito S. Wakamatsu K. (2008). Chemistry of mixed melanogenesis - pivotal roles of dopaquinone. Photochem. Photobiol. 84, 582–592. 10.1111/j.1751-1097.2007.00238.x 18435614

[B21] Ito S. Wakamatsu K. d'Ischia M. Napolitano A. P. (2011). “Structure of melanins,” in Melanins and Melanosomes: Biosynthesis, Biogenesis, Physiological, and Pathological Functions, eds Riley J. Borovansky P. A. (Weinheim: Wiley-VCH Verlag GmbH), 167-181. Available at: https://onlinelibrary.wiley.com/doi/pdf/10.1002/9783527636150#page=185 (accessed October 7, 2020).

[B22] Lauceri R. Fasciglione G. F. D'Urso A. Marini S. Purrello R. Coletta M. (2008). Kinetic investigation of porphyrin interaction with chiral templates reveals unexpected features of the induction and self-propagation mechanism of chiral memory. J. Am. Chem. Soc. 130, 10476–10477. 10.1021/ja803426q 18636730

[B23] Mammana A. D'Urso A. Lauceri R. Purrello R. (2007). Switching off and on the supramolecular chiral memory in porphyrin assemblies. J. Am. Chem. Soc. 129, 8062–8063. 10.1021/ja071447b 17552525

[B24] Marchiosi R. Soares A. R. Abrahão J. dos Santos W. D. Ferrarese-Filho O. (2020). “L-DOPA and dopamine in plant metabolism,” in Neurotransmitters in Plant Signaling and Communication. Signaling and Communication in Plants, eds Baluška F. Mukherjee S. Ramakrishna A. (Cham: Springer), 141–167.

[B25] Mason H. S. Wright C. I. (1949). The chemistry of melanin; oxidation of dihydroxyphenylalanine by tyrosinase. J. Biol. Chem. 180, 235–247. 18133389

[B26] Micali N. Vybornyi M. Mineo P. Khorev O. Häner R. Villari V. (2015). Hydrodynamic and thermophoretic effects on the supramolecular chirality of pyrene-derived nanosheets. Chem. Eur. J. 21, 9505–9513. 10.1002/chem.201500932 26012534

[B27] Occhiuto I. G. Samperi M. Trapani M. De Luca G. Romeo A. Pasternack R. F.. (2015). Aggregates of a cationic porphyrin as supramolecular probes for biopolymers. J. Inorg. Biochem. 153, 361–366. 10.1016/j.jinorgbio.2015.09.013 26490712

[B28] Onouchi H. Miyagawa T. Morino K. Yashima E. (2006). Assisted formation of chiral porphyrin homoaggregates by an induced helical poly(phenylacetylene) template and their chiral memory. Angew. Chem. 118, 2441–2444. 10.1002/ange.200504162 16526086

[B29] Panzella L. Ebato A. Napolitano A. Koike K. (2018). The late stages of melanogenesis: exploring the chemical facets and the application opportunities. Int. J. Mol. Sci. 19, 1753. 10.3390/ijms19061753 29899264 PMC6032422

[B30] Pezzella A. Napolitano A. D'Ischia M. Prota G. (1996). Oxidative polymerisation of 5,6-dihydroxyindole-2-carboxylic acid to melanin: a new insight. Tetrahedron 52, 7913–7920. 10.1016/0040-4020(96)00362-6

[B31] Pezzella A. Vogna D. Prota G. (2003). Synthesis of optically active tetrameric melanin intermediates by oxidation of the melanogenic precursor 5,6-dihydroxyindole-2-carboxylic acid under biomimetic conditions. Tetrahedron Asymmetry 14, 1133–1140. 10.1016/S0957-4166(03)00156-3

[B32] Prota G. (1995). “The chemistry of melanins and melanogenesis,” in Fortschritte der Chemie organischer Naturstoffe. Progress in the Chemistry of Organic Natural Products. Progrès Dans la Chimie des Substances Organiques Naturelles,eds W. Herz, G.W. Kirby, R.E. Moore, W. Steglich, C. Tamm (Vienna: Springer), 93–148. 10.1007/978-3-7091-9337-2_2 7782013

[B33] Rananaware A. La D. D. Al Kobaisi M. Bhosale R. S. Bhosale S. V. (2016). Controlled chiral supramolecular assemblies of water soluble achiral porphyrins induced by chiral counterions. Chem. Commun. 52, 10253–10256. 10.1039/C6CC04427A 27464524

[B34] Randazzo R. Gaeta M. Gangemi C. M. A. Fragal,à M. E. Purrello R. D'Urso A. (2019). Chiral recognition of L- and D-amino acid by porphyrin supramolecular aggregates. Molecules 24, 84. 10.3390/molecules24010084 30591641 PMC6337589

[B35] Raper H. S. (1927). The tyrosinase-tyrosine reaction: production from tyrosine of 5: 6-dihydroxyindole and 5:6-dihydroxyindole-2-carboxylic acid-the precursors of melanin. Biochem. J. 21, 89–96. 10.1042/bj0210089 16743827 PMC1251877

[B36] Ryu J. H. Messersmith P. B. Lee H. (2018). Polydopamine surface chemistry: a decade of discovery. ACS Appl. Mater. Interfaces 10, 7523–7540. 10.1021/acsami.7b19865 29465221 PMC6320233

[B37] Simon J. D. Peles D. Wakamatsu K. Ito S. (2009). Current challenges in understanding melanogenesis: bridging chemistry, biological control, morphology, and function. Pigment Cell Melanoma Res. 22, 563–579. 10.1111/j.1755-148X.2009.00610.x 19627559

[B38] Slominski A. Tobin D. J. Shibahara S. Wortsman J. (2004). Melanin pigmentation in mammalian skin and its hormonal regulation. Physiol. Rev. 84, 1155–1228. 10.1152/physrev.00044.2003 15383650

[B39] Slominski A. Zmijewski M. A. Pawelek J. (2012). L-tyrosine and L-dihydroxyphenylalanine as hormone-like regulators of melanocyte functions. Pigment Cell Melanoma Res. 25, 14–27. 10.1111/j.1755-148X.2011.00898.x 21834848 PMC3242935

[B40] Toyofuku K. Alam M. A. Tsuda A. Fujita N. Sakamoto S. Yamaguchi K.. (2007). Amplified chiral transformation through helical assembly. Angew. Chemie - Int. Ed. 46, 6476–6480. 10.1002/anie.200701668 17634993

[B41] Uemori Y. Munakata H. Kitazawa S. Osada A. Imai H. (2012). Optically active J-aggregate formed from water-soluble porphyrin with phenylalanine. J. Porphyr. Phthalocyanines 16, 1285–1292. 10.1142/S1088424612501441

[B42] Ustrnul L. Kaabel S. Burankova T. Martõnova J. Adamson J. Konrad N.. (2019). Supramolecular chirogenesis in zinc porphyrins by enantiopure hemicucurbit[n] urils (n = 6, 8). Chem. Commun. 55, 14434–14437. 10.1039/C9CC07150D 31737875

[B43] Zeng L. He Y. Dai Z. Wang J. Cao Q. Zhang Y. (2009). Chiral induction, memory, and amplification in porphyrin homoaggregates based on electrostatic interactions. Chemphyschem 10, 954–962. 10.1002/cphc.200800810 19263451

[B44] Zhao L. Liu M. Li S. Li A. An H. Ye H. (2015). Aggregation and supramolecular chirality of 5,10,15,20-tetrakis-(4-sulfonatophenyl)-porphyrin on an achiral poly(2-(dimethylamino)ethyl methylacrylate)-grafted ethylene-vinyl alcohol membrane. J. Mater. Chem. C 3, 3650–3658. 10.1039/C5TC00037H

