# Supplementary material for: En Route to a Chiral Melanin: The Dynamic “From-Imprinted-to-Template” Supramolecular Role of Porphyrin Hetero-Aggregates During the Oxidative Polymerization of L-DOPA
Source: Front Chem. 2020 Dec 21;8:616961. doi: 10.3389/fchem.2020.616961 (PMC7779627; doi:10.3389/fchem.2020.616961)
Supplement: Supplementary file 1 [file Data_Sheet_1.PDF]

Supplementary Material

En route to a chiral melanin: the dynamic “from-imprinted-to-template” supramolecular role of porphyrin hetero-aggregates during the oxidative polymerization of L-DOPA.

Massimiliano Gaeta[1], Rosalba Randazzo,[1] Valentina Villari,[2] Norberto Micali,[2] Alessandro Pezzella,[3] Roberto Purrello,[1] Marco d’Ischia,[4*] Alessandro D’Urso[1*]

[1]Dipartimento di Scienze Chimiche, Università degli Studi di Catania, Catania, Italy

[2]CNR-IPCF Istituto per i Processi Chimico-Fisici, Messina, Italy

[3]Department of Physics "Ettore Pancini", University of Naples "Federico II", Naples, Italy

[4]Department of Chemical Sciences, University of Naples “Federico II”, Naples, Italy

*** Correspondence:**
Alessandro D’Urso, adurso@unict.it

Marco d’Ischia, dischia@unina.it

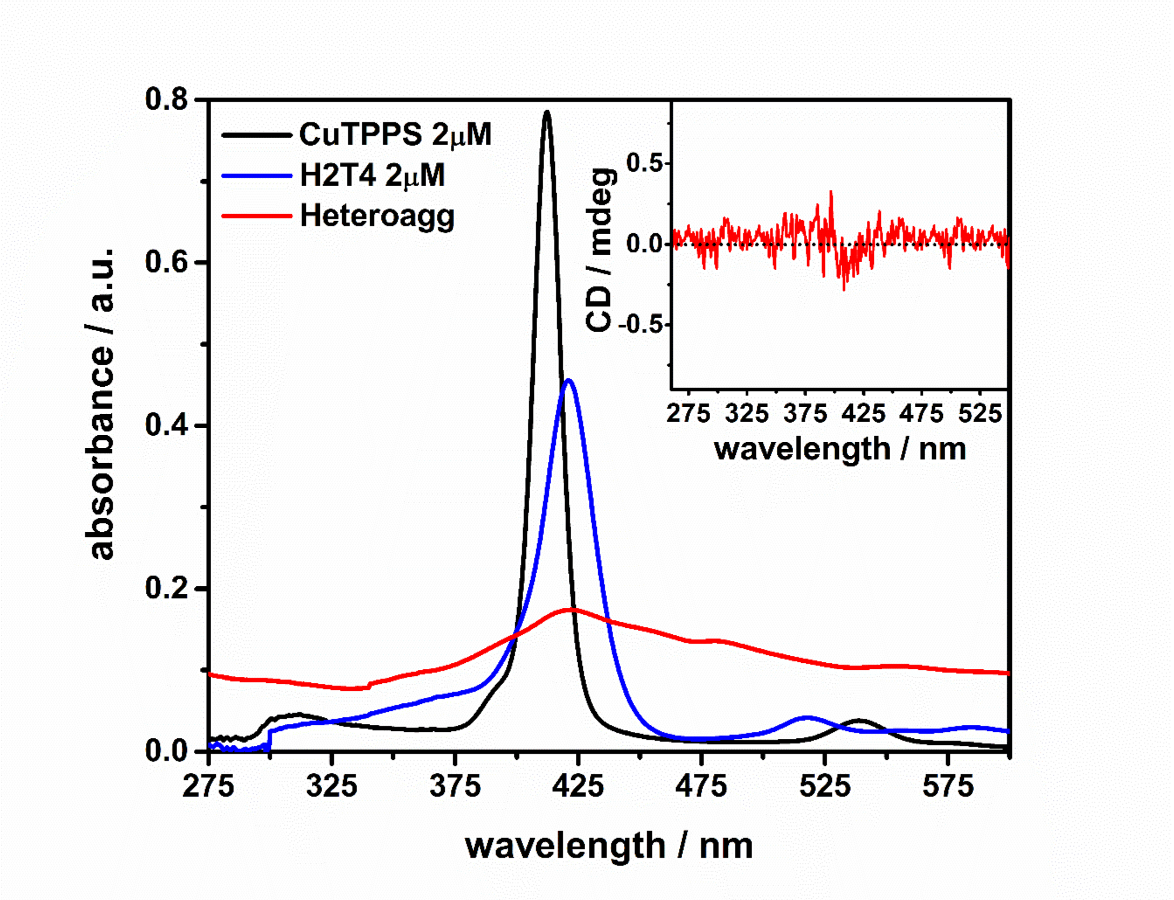

Figure S1 – UV/Vis spectra in PBS buffer (pH = 7.4) of CuTPPS alone (black curve, [CuTPPS]=2μM), H_2T4 alone (blue curve, [H_2T4]=2μM) and corresponding hetero-aggregates ([H_2T4]=4μM, [CuTPPS]=4μM). In inset is reported the CD spectrum of the same porphyrin hetero-aggregates in PBS buffer.

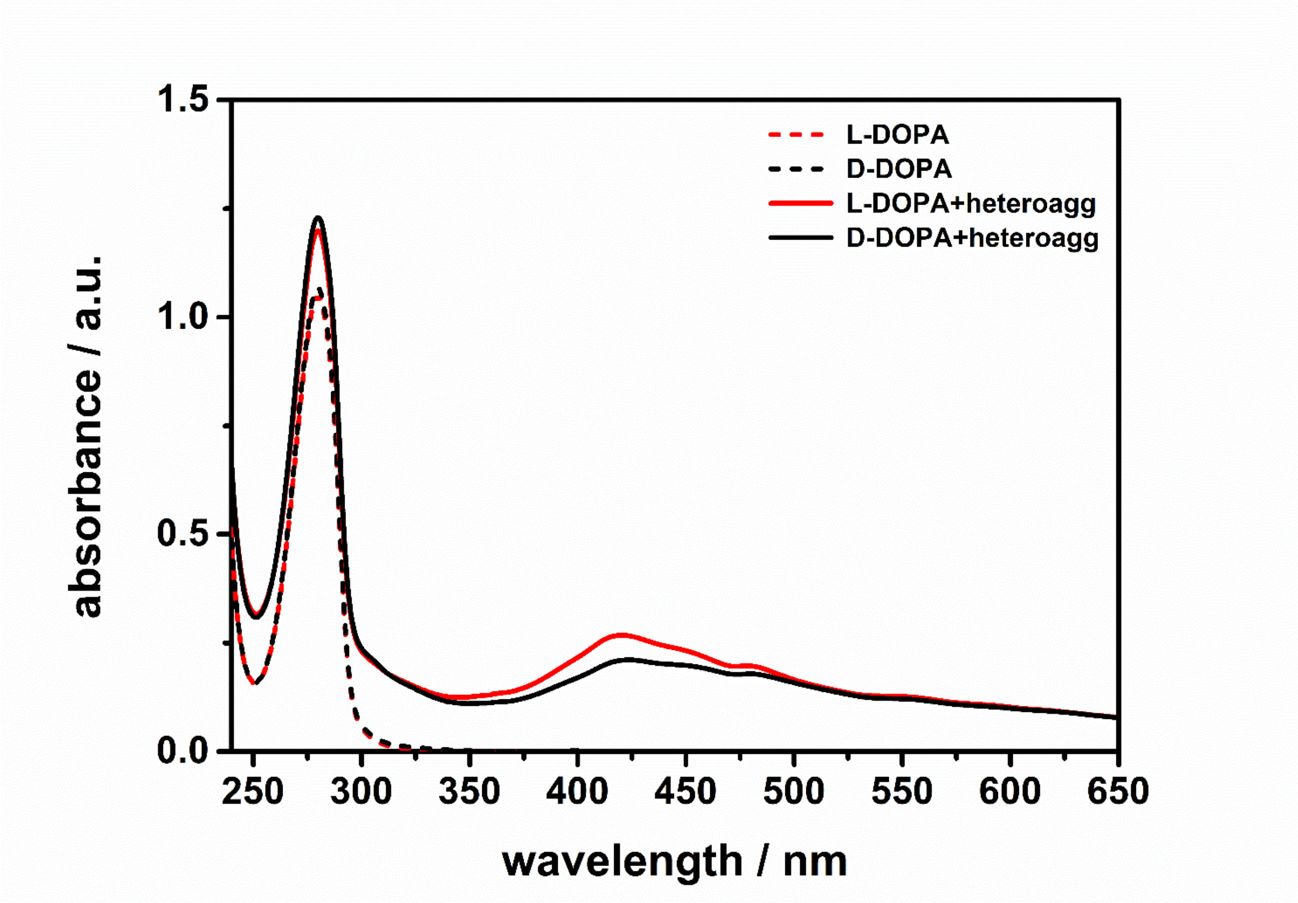

Figure S2 – UV/Vis spectra in PBS buffer (pH=7.4) of porphyrin hetero-aggregates ([H_2T4]= 4 μM, [CuTPPS]= 4 μM) in the presence of L-DOPA (red solid curve) and D-DOPA (black solid curve) as prepared. The UV/Vis spectra for DOPA alone in PBS buffer are graphed in red dashed curve, for L-enantiomers, and in black dashed curve, for D-enantiomer. In all samples the concentration of DOPA was 0.5mM.

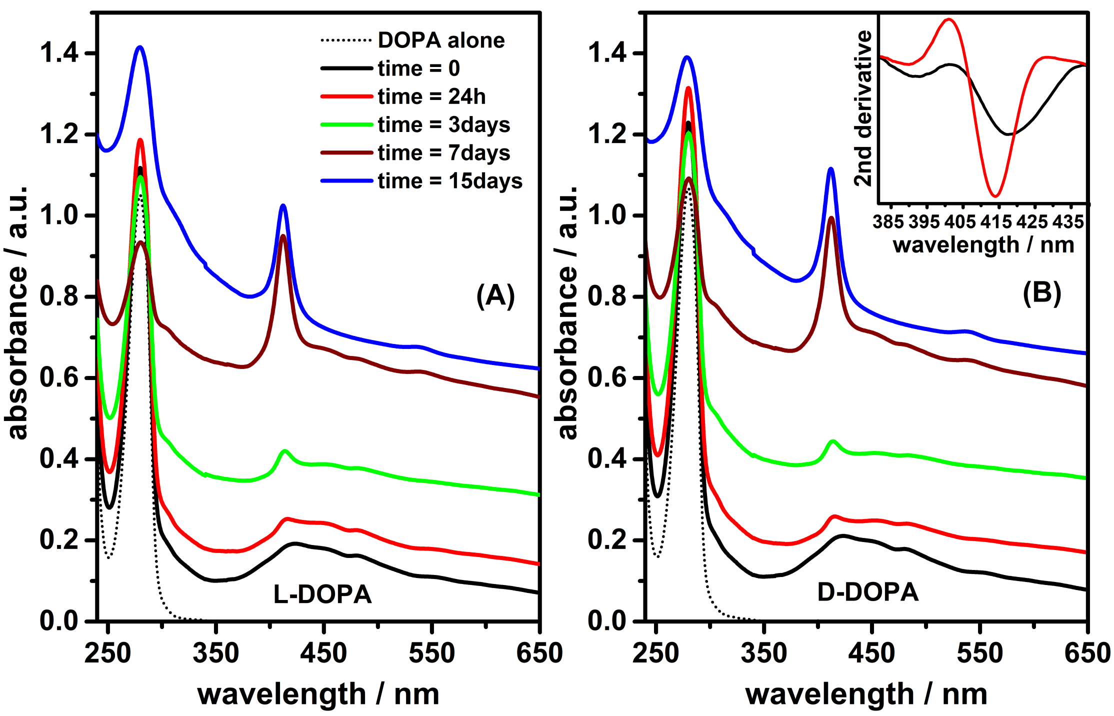

Figure S3 – UV/Vis spectra of solutions (in PBS buffer, pH=7.4) containing porphyrin hetero-aggregates ([H_2T4]= 4 µM, [CuTPPS]= 4 µM) in the presence of L-DOPA 0.5mM (A) and D-DOPA 0.5mM (B) as prepared (black curves) and after 24h, 3, 7 and 15 days (red, green, wine and blue curves respectively). As comparison, in both panels dotted curves indicate the UV/Vis spectra of L- and D-DOPA 0.5mM. Inset reports the second derivatives of sample solution as prepared (black curve) and after 24h (red curve).

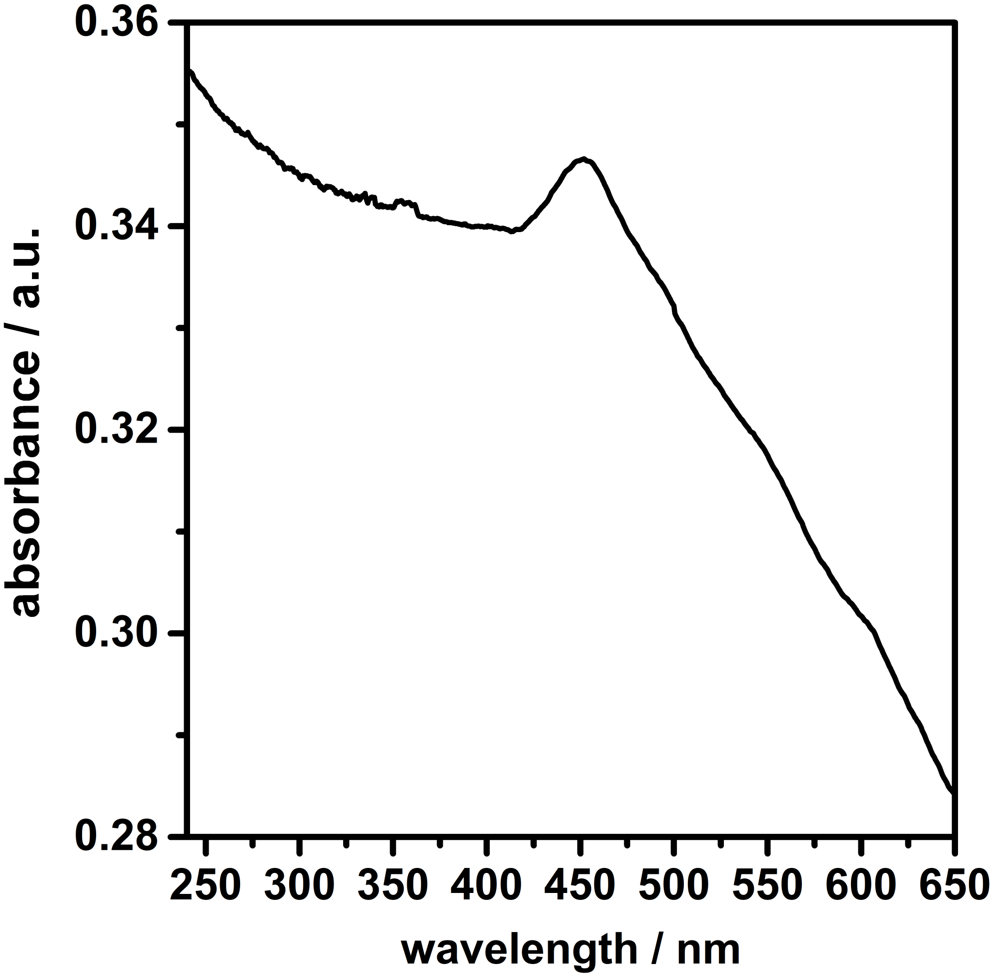

Figure S4 – UV/Vis spectra of melanin precipitate in HCl 1M.

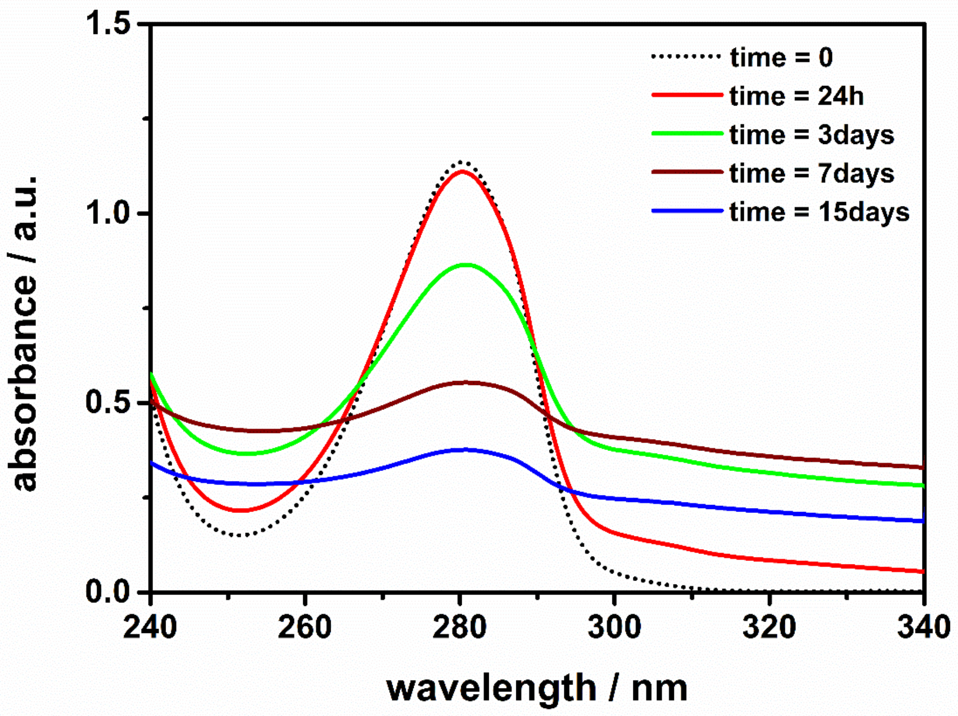

Figure S5 – UV/Vis spectra of L-DOPA ([L-DOPA]=0.5mM) alone in PBS buffer (pH=7.4) as prepared (dotted black curves) and after 24h, 3, 7 and 15 days (red, green, wine and blue curves respectively).

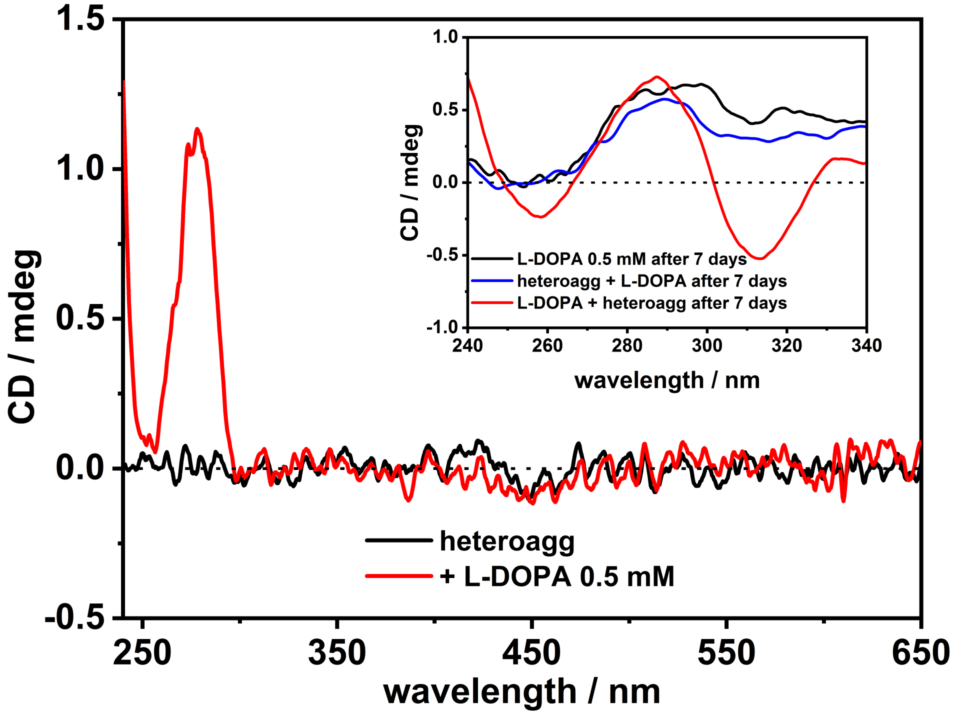

Figure S6 – CD spectra of porphyrin hetero-aggregate ([H_2T4]= 4 µM, [CuTPPS]= 4 µM) in PBS buffer, pH=7.4 before (black curve) and after the addition of L-DOPA 0.5mM (red curve). Inset reports the comparison between CD spectra of L-DOPA 0.5mM after 7 days, alone in PBS buffer (black curves) in the presence of porphyrin hetero-aggregate (red curve) and when it is added to preformed porphyrin hetero-aggregate (blue curve).
